# miRNAs in the Beta Cell—Friends or Foes?

**DOI:** 10.1210/endocr/bqad040

**Published:** 2023-03-04

**Authors:** Alexandros Karagiannopoulos, Elaine Cowan, Lena Eliasson

**Affiliations:** Department of Clinical Sciences in Malmö, Unit of Islet Cell Exocytosis, Lund University Diabetes Centre, Lund University, Skåne University Hospital, 205 02 Malmö, Sweden; Department of Clinical Sciences in Malmö, Unit of Islet Cell Exocytosis, Lund University Diabetes Centre, Lund University, Skåne University Hospital, 205 02 Malmö, Sweden; Department of Clinical Sciences in Malmö, Unit of Islet Cell Exocytosis, Lund University Diabetes Centre, Lund University, Skåne University Hospital, 205 02 Malmö, Sweden

**Keywords:** microRNA, β-cell, diabetes, insulin secretion, miR-200, LNA knockdown

## Abstract

Type 2 diabetes (T2D) develops due to insulin resistance and an inability of the pancreatic β-cells to increase secretion of insulin and reduce elevated blood glucose levels. Diminished β-cell function and mass have been implicated in impaired β-cell secretory capacity and several microRNAs (miRNAs) have been reported to be involved in regulating β-cell processes. We believe miRNAs are nodes in important miRNA-mRNA networks regulating β-cell function and that miRNAs therefore can be targets for the treatment of T2D. MicroRNAs are short (≈19-23 nucleotides [nt]) endogenous noncoding RNAs which regulate gene expression by directly binding to the mRNA of their target genes. Under normal circumstances, miRNAs act as rheostats to keep expression of their gene targets at optimal levels for different β-cell outputs. In T2D, levels of some miRNAs are altered as part of the compensatory mechanism to improve insulin secretion. Other miRNAs are differentially expressed as part of the process of T2D pathogenesis, which results in reduced insulin secretion and increased blood glucose. In this review, we present recent findings concerning miRNAs in islets and in insulin-secreting cells, and their differential expression in diabetes, with a specific focus on miRNAs involved in β-cell apoptosis/proliferation and glucose-stimulated insulin secretion. We present thoughts around miRNA-mRNA networks and miRNAs as both therapeutic targets to improve insulin secretion and as circulating biomarkers of diabetes. Overall, we hope to convince you that miRNAs in β-cells are essential for regulating β-cell function and can in the future be of clinical use in the treatment and/or prevention of diabetes.

The endocrine part of the pancreas, which constitutes 1 to 2% of the organ, is made up of 1 to 2 million cell clumps called the islets of Langerhans. These islets are ∼100 µm in diameter and contain ∼1500 cells, of which the majority are β-cells secreting insulin (1). Insulin is the only glucose-lowering hormone in the body and is released from the β-cells upon increased blood glucose levels after a meal. The inability of the β-cells to release enough insulin to lower blood glucose results in development of type 2 diabetes (T2D). Hence, the β-cells need a system for rapid and network-based regulation. MicroRNA (miRNA) networks could well play this role. MiRNAs are small noncoding RNAs regulating posttranscriptional RNA silencing and gene expression. In 2004, miR-375 was discovered as the first particularly abundant miRNA in β-cells (2). Since then, the involvement of miRNAs in regulating β-cell function, development, apoptosis, and proliferation is increasingly well documented, and changes in miRNA expression in islet cells play a key role in diabetes development (3‐8). Our knowledge so far suggests that miRNAs have both positive and negative effects on β-cell function. Therefore, the changes in miRNA expression in diabetes development could occur to compensate for or to contribute to T2D pathogenesis (4). It is also apparent that miRNAs form functional networks that orchestrate changes in gene expression to levels that are optimal for ideal β-cell function, and that this partly fails in T2D (3).

In this review, we will focus on miRNAs in pancreatic islets which have been suggested to be involved in the pathogenesis of human T2D. Here we present how miRNA networks could be best evaluated and we elaborate on the use of miRNAs as biomarkers for T2D disease and on their roles in the crosstalk between diabetes-relevant tissues. Ultimately, we promote miRNAs as excellent targets for enhancing β-cell function in T2D.

## Current Knowledge of miRNA-mRNA Interactions

In canonical miRNA biogenesis and action, the primary miRNA (pri-miRNA) of ∼1000 nucleotides (nt) in the nucleus is transcribed, spliced, capped, and poly-adenylated before it is cleaved by the Drosha enzyme to produce a 65-nt hairpin structure known as a precursor miRNA (pre-miRNA). This pre-miRNA is then exported to the cytoplasm by Exportin-5 and cleaved by the Dicer enzyme to produce a mature miRNA in the form of a small ∼22-nt RNA duplex. The mature miRNA establishes a RNA-induced silencing complex (RISC) with members of the Argonaute (AGO) protein family where it is unwound, the “passenger” strand is discarded, and the “guide” strand is retained. RISC then binds to target mRNA(s) with which the miRNAs guide strand shares perfect complementarity of 7-8 nucleotides. This 7- to 8-nt part of the miRNA is known as its seed sequence. Finally, gene and protein silencing occur via 3′end de-adenylation, degradation, or translational arrest of the target mRNAs. Different miRNAs are classified into miRNA families by virtue of having the same seed sequence, and family members often derive from a common ancestor and have similar functions even though they may differ in primary sequence or secondary structure (3, 6, 9, 10).

It has however become ever clearer that miRNA action is much more complex than described canonically above. In addition to the miRNA seed sequence, the remainder of the miRNA sequence can also dictate likelihood of mRNA target binding, a single miRNA can regulate several target mRNAs while a single target mRNA can be regulated by several miRNAs. MicroRNAs have also been reported to be affected by genetic polymorphism, promotor methylation, and RNA editing and to interact with RNA binding proteins (RBP) and other coding or noncoding RNAs (9, 11, 12). Furthermore, while the 5´ strand of the mature miRNA is usually the guide strand and miRNA binding generally occurs at the 3´UTR of the target gene(s), the 3′ miRNA strand can instead be retained by the RISC and binding can occur elsewhere, for example, at the promoter of the target mRNAs. Cell type and cellular environment thus greatly influence and regulate miRNA activity. As such, it is thus not surprising that there are now numerous studies reporting miRNA-mediated upregulation as well as downregulation of mRNA targets (12, 13).

## Literature Search

**Figure 1.**
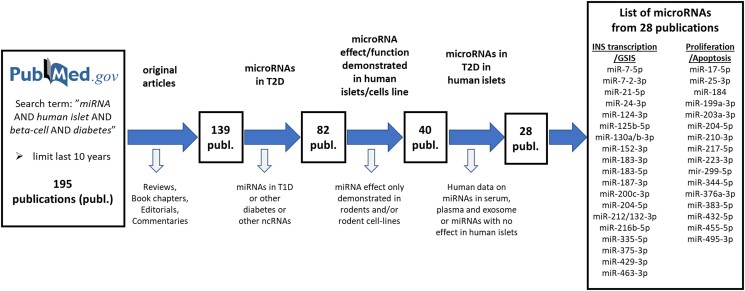
Schematic of literature search to identify relevant miRNAs investigated in human islets and coupled to T2D. A PubMed search with the search term “*microRNA* AND *human islets* AND *beta-cell* AND *diabetes*” was performed with a time limit set to 10 years, and an initial total of 195 publications were reduced in a stepwise fashion to a final 28 publications. A complete list of miRNAs reported to be involved in human islet insulin (INS) transcription, glucose-stimulated insulin secretion (GSIS), proliferation or apoptosis, and coupled to T2D over the past 10 years are detailed (see Table 1 for further details).

The current status of the field was evaluated through a PubMed search with the search term “*microRNA* AND *human islets* AND *beta-cell* AND *diabetes*” and a time limit of 10 years (Fig. 1). This yielded in total 195 publications that were carefully evaluated to find miRNAs that had been investigated in human islets and were coupled to T2D. Of these 195 publications 28 had specific focus on miRNAs in T2D in human islets. The list of the 28 publications can be found in Table 1, with documented miRNA nomenclature in the original publication and the corresponding nomenclature in the current version of miRBase (v22). At least for the oldest publications in the list, the nomenclature has been modified: current miRNA nomenclature is more specific and further denotes the functional arm of the respective miRNA (−3/5p). The table also contains information about *in vitro* and *in vivo* models that have been used, as well as validated miRNA gene targets in the respective studies. The function of the miRNA(s) is categorized by its ability to either affect apoptosis, proliferation, or glucose-stimulated insulin secretion in the β-cell. A large proportion, 56 articles, were reviews or book chapters, reflecting how much has already been summarized in this field several times. Since we included the general search term “*diabetes,*” another large proportion, 54 articles, were coupled to type 1 diabetes (T1D) (Fig. 1).

**Table 1.** Publications relevant to miRNAs in T2D in human islets

Publications	miRNA names in publication	miRNA names miRBase v22	Gene targets	Relevance to human islets	GSIS/Proliferation/Apoptosis/Other	*In vitro* model	*In vivo* model
Proliferation/Apoptosis							
PMID: 24374217	**miRNAs in the Chr 14q32 cluster incl miR-495, miR-432, and miR-376a**	**miR-495-3p, miR-432-5p, miR-376a-3p**	**TP53INP1, IAPP**	Reduced expression in T2D human islets	Apoptosis	HEK293FT cells miR-495↓ –> TP53INP1↑	
PMID: 24361012	**miR-184**	**miR-184**	**AGO2, CADM1**	Reduced expression in T2D human islets	Proliferation	MIN6 cells Key experiments to complement the experiments of *in vivo* mouse models	ob/ob, db/db and HFD-C57Bl mice –> miR-184↓ miR-184KO mice –> beta-cell mass↑ dox-Ago2 mice –> beta cell mass↑ Tg miR184 mice –> beta-cell mass↓
PMID: 31118273	**miR-223**	**miR-223-3p**	**FOXO1, SOX6**	Increased expression in T2D human islets	Proliferation	MIN6 cells i) TNFα –> miR-223↑ ii) OE of miR-223 –> proliferation↑ iii) KD of miR-223 –> proliferation↓	1 Ob/ob mice and HFD mice –> miR-223↑ miR-223 KO mice –> proliferation↓ when challenged with HFD or STZ miR-223 KO –> Foxo1↑ and Pdx1↓
PMID: 34394002	**miR-344-5p**	**miR-344-5p**	**CAV1**	Reduced expression in T2D human islets	Apoptosis	INS1 cells i) OE of miR-344 –> viability↑ and apoptosis↓ ii) KD of miR-344 –> viability↓ and apoptosis ↑	
PMID: 35906204	**miR-184-3p**	**miR-184**	**CRTC1,** **NKX6.1 (upstream)**	Reduced expression in T2D human islets	Proliferation	EndoC-βH1 KD of miR-184-3p –> protective role in palmitate- or cytokine- induced apoptosis	
PMID: 26858253	**miR-17**	**miR-17-5p**	**IFNγ (upstream)**	Reduced expression by IFNg in human islets	Apoptosis	INS1 cells i) IFNγ –> TXNIP↑ and miR-17↓ –> apoptosis↑ ii) KD of miR-17 –> IFNγ↑	
PMID: 30131392	**miR-299-5p**	**miR-299-5p**	**PERP**	Reduced expression by palmitate in human islets	Apoptosis	β-TC-6, MIN6, primary mouse islets (mouse); INS1 cells, primary SD rat islets cells (rat) KD of miR-299-5p –> β-cell function, viability↓ and apoptosis ↑	db/db mice, GK rats –> miR-299-5p
PMID: 35194770	**miR-25**	**miR-25-3p**	**MCL1, NEUROD1**	Increased expression by palmitate in human islets	Apoptosis	INS1 cells, MIN6 cells OE of miR-25 insulin content, cell viability, Ins1, Ins2, Pdx1, Gck↓ and apoptosis↑	
PMID: 35029277	**miR-455**	**miR-455-5p**	**CPEB1, EGR2 (upstream)**	Increased expression by palmitate in human islets	Proliferation	INS1 cells i) OE of miR-344 –> viability↑ and apoptosis↓ ii) KD miR-344 –> viability↓ and apoptosis↑	ob/ob mice i) small RNA sequencing –> miR-455↑ ii) increasing age –> miR-455↑ 3) injection of antimiR-455 –> mild hyperglycemia and proliferation↓
PMID: 27384111	**miR-204**	**miR-204-5p**	**PERK**	Increased expression in human islets	Apoptosis	Human islets, mouse islets, INS1 cells OE of miR-204 PERK↓ and other components in the PERK-signaling pathway INS1 cells i) KD of miR-204 PERK↑ ii) OE of miR204 –> apoptosis↑	
PMID: 23842730	**miR-199, miR-210, miR-184, miR-203, miR-132, miR-383**	**miR-199a-3p, miR-210-3p, miR-184, miR-203a-3p, miR-132-3p, miR-383-5p**	**miR-132-3p –> MAFA**	Differential expression of these miRNAs affects apoptosis and proliferation in human islets	Apoptosis/proliferation	db/db mice, HFD mice DE miRNA expression in glucolipotoxic conditions Rat islets i) OE of miR-199a –> insulin content↓ ii) OE of miR-132 –> GSIS↑ and proliferation↑ MIN6B1 cells OE of miR-132 and KD of miR-184, miR-203 and miR-383 leads to increased proliferation Human islets, Rat islets, MIN6B1 cells OE of miR-199a-3p or KD of miR-203, miR-210, and miR-383 –> apoptosis↑	
**INS transcription/GSIS**							
PMID: 24149837	**miR-187**	**miR-187-3p**	**HIPK3**	Increased expression in T2D human islets	GSIS	Rat islets OE of miR-187 –> GSIS↓	
PMID: 24789908	**miR-7a**	**miR-7-5p**	**SNCA**	Reduced expression in T2D human islets	GSIS	Mouse islets OE of miR-7a –> beta-cell exocytosis↓ Human and mouse islets KD of miR-7a –> exocytosis↑	β-cell–specific miR-7a loss-of-function mouse models –> glucose tolerance↑ and GSIS↑
PMID: 25408296	**miR-124**	**miR-124-3p**	**FOXA2, MPTN**	Increased expression in T2D human islets	GSIS	MIN6 pseudo-islets OE of miR124a –> GSIS↓	
PMID: 27664094	**miR-463-3p**	**miR-463-3p**	**ABCG4**	Increased expression in T2D donors	GSIS	MIN6 cells i)OE of miR-463 –> GSIS↓ ii) KD of miR-463 –> GSIS↑	
PMID: 28332581	**miR-130a/b, miR-152**	**miR-130a-3p, miR-130b-3p, miR-152-3p**	**PDHA1**	Increased expression in T2D human islets	GSIS	INS1 cells i) OE of any of the miR-130a/b, miR-152 reduces GSIS and ATP levels ii) KD of any of the miR-130a/b, miR-152 increases GSIS	GK-rat islets miR-130a/b, miR-152↑
PMID: 29122960	**miR-335**	**miR-335-5p**	**SNAP25, STXBP1, SYT11**	Negative correlation with GSIS in human islets from donors with prediabetes	GSIS	INS1 cells i) OE of miR-335 –> GSIS↓ and exocytosis↓ ii) KD of miR-335 –> exocytosis ↑	
PMID: 34753799	**miR-200**	**miR-20 0c-3p**	**ETV5**	Increased expression in T2D human islets	GSIS	EndoC cells OE of miR200c –> GSIS↓ Human islets KD of miR200c in T2D islets –> GSIS↑	
PMID: 23761103	**miR-24**	**miR-24-3p**	**NEUROD1, HFN1α**	Increased expression by palmitate in human islets	GSIS	MIN6 cells i) OE of miR-24 –> GSIS↓ and proliferation↓ ii) KD of miRNA-24 targets –> GSIS↓ and proliferation↓ HFD mouse islets KD of miR-24 –> GSIS↑	db/db mouse islets –> miR-34, miR-146, miR-37a, and miR-24↑ and miR-21, miR-30c, miR-7, miR-127, and miR-375↓
PMID: 34246804	**miR-21**	**miR-21-5p**	**TGFB2, SMAD2**	OE in human islets –> PDX-1, TGFB2 and SMAD2↓ and Aldh1a3↑ –> dedifferentiation	GSIS/beta-cell mass	INS1 cells OE of miR-21 –> GSIS↓, proinsulin↑ Tg(βmiR-21) mouse islets β-cell–specific OE of miR-21 –> insulin secretion↓	Tg(βmiR-21) mice blood glucose during IPGTT↑ and β-cell identity↓
PMID: 26218441	**miR-212/132**	**miR-212-3p, miR-132-3p**		Expression is induced by GLP-1 in human islets	GSIS	INS1 cells i) OE of miR-132 –> GSIS↑ with or without the presence of GLP-1 ii) KD of miR-132 –> no effect	Exendin-4 (GLP-1 analog) infused C57BL/6N mice –> miR132/212↑ in the islets
PMID: 35476777	**miR-125b**	**miR-125b-5p**	**M6PR, MTFP1**	Increased expression by glucose in human islets. Positive correlation with BMI	GSIS	MIN6, EndoC cells KD of miR-125b –> GSIS↑ MIN6 cells OE of miR-125b –> GSIS↓	TgmiR125b mouse i) blood glucose during IPGTT↑ ii) Islets –> GSIS, KSIS, number of docked granules↓ and defective mitochondria and lysosomes
PMID: 23975026	**miR-204**	**miR-204-5p**	**MAFA, TXNIP (upstream)**	OE –> insulin mRNA↓ in human islets	GSIS	INS1 cells OE of miR-204 –> *INS* expression, INS content, GSIS↓ Human islets OE –> *INS* expression↓	
PMID: 29101219	**miR-204**	**miR-204-5p**	**GLP1R**	OE –> GLP1R↓ in human islets	GSIS	INS1 cells i) OE of miR-204 –> GLP1R↓ ii) KD of miR-204 –> GLP1R↑ Human and mouse islets OE of miR-204 –> GLP1R↓	miR204 KO mice i) Blood glucose in GTT↓ ii) GSIS↓ with or without Exendin-4 β-Cell–Specific TXNIP Knockout Mice i) miR-204↓ and GLP1↑ ii) blood glucose during GTT↓ in presence of Exendin-4
PMID: 29070792	**miR-204**	**miR-204-5p**		Highly enriched in human islets, EndoC, and beta-cell-like iPSCs	GSIS –> no effect (see earlier work by Shalev; PMID: 23975026)	Human islet, EndoC cells KO of miR-204 and OE of miR-204 –> no effect on insulin secretion, *MAFA, INS, TXNIP, TRMP3* expression	
PMID: 29269398	**miR-184**	**miR-184**	**AMPK (upstream)**	OE of miR-184 in human islets; not DE in human T2D islets; DE between male and female human islets	Investigation of the regulation of miR-184 in human islets	Human islets, MIN6 cells AMPK-activators –> miR-184↑	AMPK-KO mice –> miR-184↓ –> GSIS↑(previously shown in PMID: 25070369)
PMID: 31697642	**miR-375**	**miR-375-3p**	**MDH1, PC**	OE of miR-375 reduced GSIS in human islets	GSIS	Rat islets i) OE of miR-375–> GSIS, glucose-stimulated cytosolic Ca2+, and oxygen consumption (ATP)↓ ii) KD of miR-375–> GSIS and oxygen consumption↑ Human islets OE of miR-375 –> GSIS↓	
PMID: 33981968	**miR-183-5p, 375-3p, 216b-5p, 183-3p, 7-5p, -217-5p, 7-2-3p, and 429-3p**	**miR-183-5p, miR-375-3p, miR-216b-5p, miR-183-3p, miR-7-5p, miR-217-5p, miR-7-2-3p, miR-429-3p**		miRNAs associated with INS transcript in human islets	GSIS; de-differentiation	microRNA overexpressing PANC cell lines using lentiviral-mediated, doxycycline-inducible overexpression system –> Confirmation of the regulatory roles of these microRNAs in (pro-)endocrine gene expression Human islets Knockdown of these microRNAs –> (pro-)insulin transcript abundance	

PUBMED search was performed with the terms “microRNA” AND “human islet” AND “beta-cell” AND “diabetes.” Relevant information from publications satisfying the filtering criteria and are focused on miRNAs in T2D in human islets are presented. “Upstream” target refers to the gene that regulates the expression of the miRNA.

Abbreviations: DE, differentially expressed; GK, Goto-Kakizaki (rats); GSIS, glucose-stimulated insulin secretion; GTT, glucose tolerance test; HFD, high-fat diet fed; IPGTT, intraperitoneal glucose tolerance test; iPSC, induced pluripotent stem cell; KD, knockdown; KO, knockout; KSIS, K^+^-stimulated insulin secretion; OE, overexpression; STZ, streptozotocin.

## Islet miRNAs Important for Human T1D

MiRNAs are important regulators of gene expression in processes important for β-cell function in human islets. From the literature search, it was made obvious that T1D miRNA studies are moving in 3 important directions. First, miRNAs are proven useful to differentiate induced pluripotent stem cells (iPSCs) or other pancreatic cells into insulin-secreting-like cells with the eventual goal of such cells being used as a resource for cell replacement therapy in T1D patients. Many have succeeded using overexpression of miR-375 and/or miR-7 (14‐17), showing the importance of miRNAs in shaping the β-cell phenotype to produce insulin-secreting cells. Second, miRNAs in exosomes and/or in blood could act as biomarkers. Exosomes are membrane-bound extracellular vesicles that can be released through exocytosis into the blood from one cell type and taken up by another. Exosomes can have specific miRNAs as cargo. One interesting hypothesis coupling miRNAs to T1D is that exosomes have been shown to contain miRNAs from inflammatory cells that transmit a signal to β-cells (18, 19). Not only in T1D, but also in T2D, exosomes are important carriers of information between cell types. Among the 195 publications in our PubMed search, 12 publications concerned T2D and exosomes (Fig. 1). We are continuously learning more about exosome content, including miRNAs. Third, other T1D studies focus on measuring miRNAs in the circulation as biomarkers for β-cell destruction (20, 21), often inspired by investigating the intracellular function of miRNAs involved in β-cell death in T1D (22, 23). In T1D development, miRNAs partake in the crosstalk between cells and in the regulation of intracellular processes leading to apoptosis, and when measured in this respect they can therefore also serve as biomarkers in blood.

## miRNAs Important for β-cell Function in T2D Development

**Figure 2.**
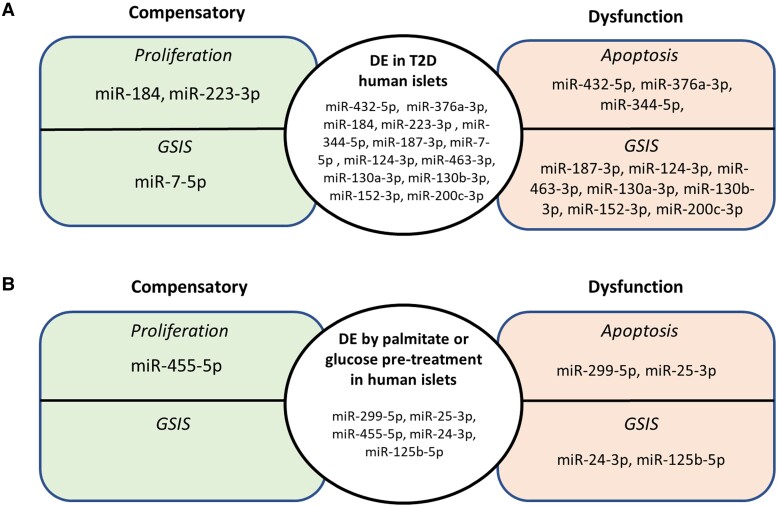
The miRNAs differentially expressed (DE) in human islets affecting compensatory responses or dysfunctional changes with T2D, or palmitate/glucose pretreatment. Changes in the expression of certain miRNAs can occur as part of the pathogenesis of disease, promote apoptosis and impair GSIS to cause islet dysfunction. Alternatively, changes in miRNA expression can occur in response to disease pathogenesis and instead play a compensatory role by enhancing proliferation and improving GSIS to combat disease progression. This figure summarizes miRNAs whose expression is altered in T2D (A), or after palmitate or glucose pretreatment of islets (B) and the compensatory (left) or dysfunctional (right) effects of these changes on islet function.

In human T2D development, differential expression of miRNAs can potentially occur either due to β-cell dysfunction or as a compensatory response mechanism to the same (Fig. 2). In our investigation of 195 publications (Fig. 1), 28 could be coupled to human islet T2D, either through differential expression between islets from nondiabetic and T2D donors, differential expression induced by palmitate, cytokine, or glucose treatment in human islets, or via investigative studies where specific miRNA expression has been manipulated in human islets (Table 1).

Several miRNAs regulate apoptosis and proliferation. MicroRNAs in the DLK-MEG cluster have reduced expression in T2D islets, and this reduction contributes to β-cell dysfunction by increased apoptosis (5). This can occur via miRNA regulation of proteins involved in the endoplasmic reticulum stress pathway. Examples are miR-17 regulating TXNIP (24) and miR-204 regulating PERK (25). Another, more recent, report of a miRNA regulating apoptosis is miR-344-5p, where reduced expression was measured in pancreatic slices from T2D donors, and where deletion experiments in INS-1 cells increased apoptosis (26). MiRNAs whose reduced expression results in increased apoptosis can be considered “foes” since they contribute to diabetes development. On the positive side, these miRNAs could also be good therapeutic targets. Differential miRNA expression inducing changes in proliferation can often have a compensatory function in the β-cell. This has been shown for miR-184 in several studies, all of which agree on a compensatory response with decreased expression of miR-184 in islets from T2D donors and the involvement of this miRNA in the regulation of proliferation (23, 27, 28), irrespective of disagreements between groups regarding the targets involved. In all, miR-184 together with miR-223-3p (29) can be regarded as compensatory miRNAs and therefore “friends” in the sense that they act to counteract diabetes development (Fig. 2).

**Figure 3.**
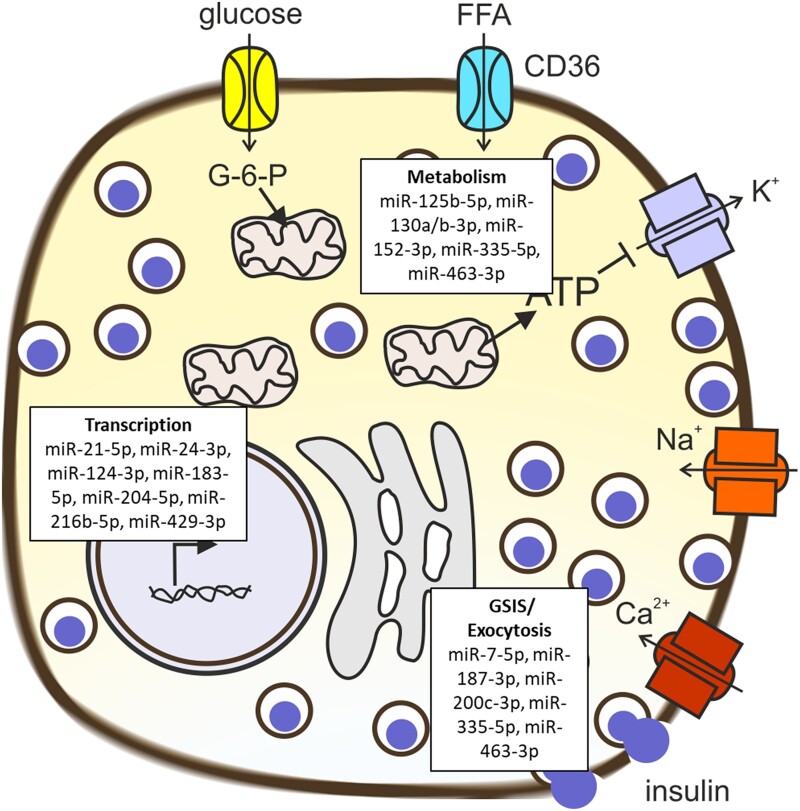
Stimulus-secretion coupling of the β-cell incorporating miRNAs in 3 different functions. Glucose is taken up from the blood through glucose transporters (GLUT) and converted via glucokinase to glucose-6-phosphate (G-6-P) which is then taken up by the mitochondria. Mitochondrial metabolism leads to the generation of ATP and an increase in ATP closes the ATP-dependent K^+^ channels leading to membrane depolarization. This in turn opens voltage-dependent Na^+^ and Ca^2+^ channels leading to an influx of Ca^2+^, which triggers exocytosis of insulin granules. Prior to exocytosis insulin needs to be transcribed from the insulin gene (INS) and translated into pre-proinsulin. Proinsulin is packed into insulin granules where it is cleaved into insulin and C-peptide. The insulin granules need to dock at the plasma membrane before Ca^2+^ dependent exocytosis can occur. Cellular function of miRNAs involved in the regulation of glucose-stimulated insulin secretion (GSIS; from Table 1) are divided into 3 different groups; 1) Transcription, either regulation of INS transcription or regulation of transcription factors, 2) Metabolism, and 3) GSIS/Exocytosis, including miRNAs affecting exocytosis or there was no specific cellular function determined only shown that GSIS was reduced.

Stimulus-secretion coupling (Fig. 3) of the β-cell describes the systematic line of mechanisms in the cell from glucose uptake to release of insulin, a process that has been well established for many years (1). Many miRNAs also affect these processes. Of the miRNAs in Table 1, the majority have a role in the regulation of glucose-stimulated insulin secretion affecting either transcription, metabolism, or exocytosis as described in Fig. 3. Among more recent findings, an important miRNA of interest is miR-125b-5p. This miRNA has increased expression after long-term incubation of human islets with high glucose (30). The transgenic miR-125-5p mouse model has increased blood glucose during an intraperitoneal glucose tolerance test (ipGTT) and islets from these mice have reduced glucose-stimulated insulin secretion, defective mitochondria and lysosomes, and a reduced number of docked granules. MicroRNA-125b-5p is regulated by AMPK and targets the mitochondrial fission regulator Mtfp1 and the transporter of lysosomal hydrolases M6pr. The latter target could suggest involvement of miR-125b-5p in autophagy. Understanding the role of miR-204-5p is however more complex. While in 3 different studies overexpression in human islets showed increased apoptosis, reduced insulin secretion, and decreased expression of glucagon-like peptide 1 (GLP-1) receptors (25, 31, 32), a more recent human islet study neither validate the gene targets nor confirm the reduction in insulin secretion (33).

Within the group of miRNAs differentially expressed in T2D islets and involved in the regulation of insulin secretion, we found increased expression of miR-187-3p, miR-124-3p, miR-463-3p, miR-130a-3p, miR-130b-3p, miR-152-3p, and miR-200c-3p (34‐38) to be involved in β-cell dysfunction, while decreased expression of miR-7-5p (miR-7a; (39)) seems to be a mechanism by which this miRNA exerts a compensatory regulatory effect on exocytosis (Fig. 2). We have specifically focused on further discussing 4 different miRNAs from Table 1 that regulate glucose-stimulated insulin secretion. In our opinion, these have potential to be good targets for the treatment of T2D in the future, and to paint as full picture as possible, we have extended our focus to dicuss all family members.

### miR-7 Family

miR-7 is a highly conserved miRNA family encoded by 3 different loci in humans and mice, and it is also highly expressed in neurons and neuroendocrine organs, including the pancreatic islets (5, 39‐41). Both miR-7a and miR-7b are expressed in the islet cells, of which miR-7a2 is the dominant form (39). MicroRNA-7 has been extensively studied as a miRNA important for β-cell development and regulation of insulin transcription (8, 41, 42) (Fig. 2). Several β-cell markers are regulated by miR-7 and the miRNA has been suggested to be involved in β-cell differentiation; for example, studies in transgenic mice overexpressing miR-7 showed reduced expression of both *Pdx1* and *Ins* (41), while miR-7 knockdown experiments in mice showed an increase in *Pax6* expression promoting α- and β-cell differentiation (42).

In addition, miR-7 regulates β-cell exocytosis through the target gene *Snca* (α-Synuclein). Overexpression of miR-7 reduced exocytosis measured by capacitance measurements in single β-cells without affecting Ca^2+^ oscillations, suggesting a role in direct interaction with exocytosis (39). Furthermore, knockdown of miR-7 improved the exocytotic response. Importantly, data from this work also showed that 1) α-Synuclein interacted with the SNARE-protein Vesicle associated membrane protein 2 (VAMP2); and 2) miR-7 regulated the stoichiometric interaction between α-Synuclein and VAMP2, and ultimately the availability of oligomeric SNARE-complexes.

Interestingly, comparing miR-7 with another abundant islet miRNA, miR-375, revealed some major differences. Even though both are highly conserved and abundant in islet cells, knockdown of miR-7 results in improved insulin secretion, whereas knockdown of miR-375 reduces β-cell mass. Moreover, miR-7 is differentially expressed in T2D islets whereas miR-375 is not. As pointed out above, data relating to miR-7 expression in T2D islets suggest that this miRNA is downregulated in T2D (5, 39), indicative of a compensatory mechanism to improve insulin transcription and secretion in the β-cell.

### miR-130a/b and miR-148/152 Families

The miR-130 family has 4 members (miR-130a, miR-130b, miR-301a, and miR-301b) and research has thus far primarily focused on cancer biology. Altered levels of miR-130a and miR-130b have, however, been reported in obesity in human studies and both miRNAs have also been suggested to play a role in insulin resistance (43‐45). In the case of the miR-148/152 family there are 3 members (miR-148a, miR-148b, and miR-152) and research has primarily focused on immunity and cancer biology (46, 47).

In islets, these 2 families were discovered to be involved in T2D pathogenesis through a miRNA profiling study from type 2 diabetic Goto-Kakizaki rats. This study revealed 24 miRNAs which were upregulated in Goto-Kakizaki islets, including miR-130a and miR-152 (48). Further follow-up (37) revealed that miR-130a, miR-130b, and miR-152 were also upregulated in either glucose-intolerant or T2D human donor islets compared with nondiabetic control islets, thereby confirming the importance of these miRNAs in human T2D biology. Mechanistic studies to investigate miR-130a/b and miR-152 effects on β-cell metabolism confirmed 2 interesting gene targets: *Gck* (glucokinase) and *Pdha1* (pyruvate dehydrogenase E1 subunit alpha 1). The model put forward suggested that upregulation of miR130a/b and/or miR-152 in β-cells leads to downregulation of GCK and PDHA1, significantly reducing β-cell cytosolic ATP:ADP ratio, insulin content, and insulin release (37). Hence, these miRNAs play a role in the regulation of ATP generation, as highlighted in Fig. 2. A later 2017 study also investigated miR-152 mechanistically in diabetes. Here, Chen *et al* conversely reported reduced miR-152 expression in T2D patients, and increased proliferation and insulin secretion after miR-152 overexpression in INS-1 and MIN-6 cells. Furthermore, they purported that these effects occur via miR-152 targeting of PI3K catalytic subunit α (PI3Kα) (49). More recent publications by other groups also reported that miR-152 targets SOCS3 to inhibit hepatic insulin resistance in mice with gestational diabetes and that miR-148 silencing in MIN-6 β-cells and mouse islets downregulates insulin promoter activity and expression (50, 51). Nevertheless, to the best of our knowledge, our work (37) comprises the most extensive mechanistic investigations of the roles of miR-130 and miR-152 in human β-cell and islet metabolism. Hence, we believe miR-130a/b and miR-152/148 are upregulated in islets in T2D, and that applying knockdown can be used as a therapeutic tool to improve insulin secretion.

### miR-200 Family

There are 5 members of the miR-200 family (miR-200a, miR-200b, miR-200c, miR-429, and miR-141) that are derived from 2 different chromosomal locations, human chromosome 1 (miR-200a, −200b, −429), and human chromosome 12 (miR-200c, and −141). Moreover, the family is divided into 2 classes (miR-200a, −141 and miR-200b, −200c, −429) based on the homology of their seed sequences, with only a single base difference between the groups (52). The miR-200 miRNAs are among the more abundant and well-studied β-cell miRNAs, with their expression being higher in β- than α-cells (53).

Recent work from our laboratory demonstrated a role for miR-200c in β-cell insulin secretion (Fig. 3) through the regulation of transcription factor ETV5 (E26 variant gene 5) (36). With the use of knockout mice, ETV5 has previously been demonstrated to have a role in exocytosis (54), and this was confirmed in human islets when we demonstrated an association between expression of ETV5 and exocytotic genes (36). We also found miR-200c to be upregulated in human islets from T2D donors and confirmed previously identified targets involved in the regulation of β-cell proliferation and apoptosis (55, 56). The miR-200 family has been reported to regulate β-cell survival in response to metabolic stress and β-cell apoptosis/proliferation has been shown to be modulated by several miR-200 gene targets, including *JazF1* (Juxtaposed with another zinc finger protein 1) and *Zeb1* (zinc finger E-box-binding homeobox 1). Of interest from a diabetic perspective, the pro-apoptotic regulator Txnip (Thioredoxin-interacting protein) has been shown to induce the expression of miR-200a, miR-200b, miR-200c, miR-141, and miR-429 (56).

Since miR-200c is upregulated in T2D islets, therapeutic knockdown is potentially of interest. Indeed, locked nucleic acid (LNA) mediated knockdown of miR-200c in human islets from T2D donors has been demonstrated to improve insulin secretion (36).

## miRNA “Networking”—Bringing the Pieces Together

Several studies have identified islet-enriched miRNAs (7, 57) and highlighted the important roles of individual miRNAs in different aspects of β-cell function (Table 1, Fig. 3) by targeting various molecular pathways including glucose metabolism, cell development, proliferation, and insulin synthesis and secretion (3, 58). However, due to their short seed sequence each miRNA can recognize the complementary sequences of multiple target genes (59), with estimates suggesting that more than half of all protein-coding genes in the human cell are regulated by miRNAs (11). This suggests that specific miRNAs can regulate several cellular processes and that one process can be regulated by multiple miRNAs; for example, miR-204 can regulate both β-cell apoptosis (via *PERK*) and glucose-stimulated insulin secretion (via *MAFA* and *TXNIP*) (Table 1) (32, 60). The importance of the collective action of miRNAs is demonstrated by genetic ablation of Dicer in the β-cells of mice, which causes defective insulin secretion and reduced β-cell mass (61, 62). Fortunately, the generation and availability of a considerable amount of data has paved the way for network-based approaches to answer complicated molecular questions (63) such as miRNA regulation.

Publicly available databases provide comprehensive lists of experimentally validated miRNA targets (64, 65), which in combination with RNA-seq data, can be used to identify robust miRNA-gene interactions. This approach has been applied to detect novel miRNA-mRNA networks involving miRNAs that are altered in a glucose-dependent manner in rat INS-1 cells (66) and/or by glycemic status in islets from human donors (5). In both cases, overlapping sets of miRNAs seem to be interacting with gene clusters involved in islet-specific functional pathways, including glucose metabolism and insulin secretion. In another study, authors have described a machine-learning method to accurately determine miRNAs associated with insulin transcription (8). They conclude that measuring a group of 8 miRNAs is adequate to define insulin-expressing tissues and predict insulin transcription levels in human islets (Table 1, Fig. 3) and pancreatic cell lines. Moreover, by integrating validated miRNA gene targets with pancreatic single-cell whole transcriptome data, they could infer miRNA-mRNA interactions important for the differentiation of endocrine pancreatic lineage (8).

Single-cell small RNA sequencing data in human islets is still limited, as just 2 studies so far have characterized the miRNA profiles of alpha and beta cells (7, 53). Although challenging due to low abundance of miRNAs and low proportion of islet cell types other than α- and β-cells (eg, pp-, δ, and *ε*-cells), it is important to identify miRNA profiles of all endocrine cell types and combine these with cell-specific mRNA expression data. This kind of analysis in other cell types has demonstrated large heterogeneity in miRNA-mRNA interactions even within cells of the same cell type (67, 68) and could thus partially explain the functional heterogeneity of individual endocrine cells of the same type (69, 70). Taking it a step further, spatial and temporal small RNA sequencing at the single-cell level could provide us with unique insights into the way miRNAs act on their targets in a spatial- and time-dependent manner. Since miRNA action appears to be controlled epigenetically (71), as well as by the effect of transcription factors in positive and negative regulatory loops (72), a multi-omics network approach seems to be necessary in order to accurately define the miRNA mode of action. Powerful computational tools will allow the integration of additional interactions that have been shown to influence miRNA activity and are mediated by long noncoding (lnc)RNAs (73) and single nucleotide polymorphisms (SNPs) (74). With this approach, we could potentially uncover further layers of complexity in the context of miRNA action in β-cells in a similar fashion to what has already been observed in other tissues, namely distinct miRNA targeting of different gene targets, multiple mechanisms of repression of given genes, and condition-specific miRNA action (75, 76). This will pave the way toward deciphering single-cell–specific causal regulatory networks by integrating high-throughput screening such as AGO immunoprecipitation sequencing (AGO IP-seq), AGO cross-linking and immunoprecipitation sequencing (CLIP-seq) and chromatin immunoprecipitation sequencing (ChIP-seq) and combining the findings thereafter with information about single-cell functional properties investigated by total RNA sequencing of endocrine cells (77). A cell type–specific miRNA-mRNA regulatory map for each cell type could then be used to identify unwanted miRNA off-target effects within one cell type or across multiple cell types via cellular crosstalk. Using the appropriate miRNA combinations, we could design new therapeutic targets for diabetes treatment with fewer off-target effects, as seems to be the case of approach for other diseases like cancer (78). Furthermore, complete cell-specific miRNA maps in combination with miRNA data from released exosomes from the different islet cells and their surrounding tissues under certain conditions (for example nondiabetic *vs* T2D, normal diet *vs* high-fat fed diet), will make it possible to fully elucidate miRNA regulation during the development of T2D, and confidently determine which miRNAs are the best biomarkers of the disease.

**Figure 4.**
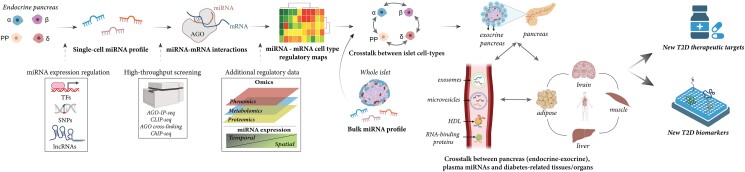
Strategy to find novel miRNA therapeutic targets and biomarkers via miRNA network integration analysis. Islets from human donors are prepared to measure miRNA expression and to build miRNA-mRNA networks connected to T2D development. This information can then be utilized to find key miRNAs for therapeutic treatment of T2D and identify biomarkers of disease.

To summarize, we believe the future of miRNA research will involve increased understanding of miRNA-mRNA networks to identify new therapeutic targets and biomarkers. In Fig. 4, we visualize our idea of a work process which includes integrating information about miRNAs at the single-cell level to all diabetes-related tissues and to circulating miRNAs either within exosomes or bound to proteins.

## Is There a Future for miRNAs as Therapeutic Targets and Biomarkers?

Knowing that miRNAs impact insulin secretion through multiple cellular pathways, it is obvious to question whether miRNAs can become therapeutic targets. Several candidates have been tested and are now in phase 1 and phase 2 clinical trials, for example, a locked nucleic acid (LNA)-based drug to inhibit miR-92 has potential in wound healing (79). Moreover, studies in T2D animal models have suggested strategies that could potentially be translated to human biology (80‐82), employing different approaches to reduce the expression of miRNAs using chemically modified antagomirs. Overall, the most positive taking from these investigations, which therefore holds promise for the future, is the finding that LNA molecules with modified backbones are more stable in blood (83). However, like any drug-development strategy, a huge challenge is tissue-specific delivery of such RNA-based therapeutics. Another factor to consider is poor uptake in the tissue. This can be overcome by using viral and synthetic delivery systems. Viral delivery of synthetic miRNAs is very efficient using adenovirus (Ad), adeno-associated virus (AAV) and lentivirus (LV) vector systems (84). To track uptake, one possibility is to also add a fluorescent marker to the viral construct.

In our hands, injection of an antagomir against miR-132 into the bloodstream of mice resulted in delivery of the antagomir into the pancreatic islets, reduction of miR-132 levels, and subsequent improvement of blood glucose levels (81). Although this is a promising “proof-of-concept” work, specific delivery into the relevant cells/tissues of RNA-based drugs is highly desired. Perhaps specialized nanoprobes could help with cell-specific delivery. The use of antagomirs or LNA-probes could potentially be an excellent choice since it is possible to silence several pathways regulated by the same miRNA with these chemistries. However, there are still many challenges with RNA-based inhibitors of miRNAs, and further development of antagomirs is needed before use. Factors to consider are specificity and off-target effects, sensitivity of inhibitors, uptake in the tissue, and target cell delivery. Another factor to consider is how miRNAs may be modulated by current treatments such as metformin and GLP-1. In a recent study, metformin was shown to increase the expression of Dicer, and thereby the expression of miRNAs (85). Moreover, GLP-1 has been demonstrated to increase expression of miR-212/132 (86), and miR-204 overexpression in human islets decreases the expression of GLP-1 receptors (31). Hence, although the concept of using miRNA inhibition offers novel treatment strategies for T2D and associated complications, there are many factors to consider. Still, miRNAs as potential therapeutic targets hold excellent promise for the future.

Identifying miRNAs as biomarkers for future clinical use is another possibility. Data from recent years have demonstrated the release of miRNA-containing exosomes into the circulation in both T1D and T2D (18, 87). Moreover, changes in blood miRNA profile can also occur due to the destruction of the β-cells. Indeed, several studies have measured the differential expression of miRNAs in disease development; for example, in a predictive study of diabetes and cardiovascular disease with >500 individuals without disease at baseline, of whom ∼30% developed diabetes during follow-up, miR-483-5p was associated with diabetes development (88). Although in diabetes, blood glucose is the best diagnostic biomarker, miR-483-5p can act as a prognostic biomarker that can detect disease-specific changes that occur prior to changes in blood glucose. One major drawback is that a specific miRNA could also be the marker of another disease. Therefore, specialized biomarker panels comprising several miRNAs together with other markers of the disease are required. Hence, a role for miRNAs as biomarkers also holds promise for the future.

## Concluding Remarks

Time to answer the question: miRNAs in the beta cell—friends or foes? Under normal circumstances miRNAs are our best friends acting as rheostats and controlling gene expression levels. They are also highly involved in differentiation and proliferation processes, and in T2D development, some miRNAs are still friends partaking in the compensatory mechanism to improve insulin secretion and increase proliferation in order to overcome hyperglycemia. Other miRNAs, however, are more like foes, merely because the differential expression of these miRNAs can underlie mechanisms causing β-cell dysfunction and thereby contribute to T2D pathogenesis. However, it is fortunate we can also take advantage of these specific miRNAs by using them as therapeutic targets. So, in our opinion, based on the current knowledge, miRNAs in the β-cell are more friends than foes.

## Data Availability

All data generated or analyzed during this study are included in this published article.

## Abbreviations

AGO: Argonaute proteins
GLP-1: glucagon-like peptide 1
LNA: locked nucleic acid
miRNA: microRNA
nt: nucleotides
RISC: RNA-induced silencing complex
T1D: type 1 diabetes
T2D: type 2 diabetes
